# Mechanistic insights into the anti-depressant effect of quercetin: an integrated bibliometrics, bioinformatics, and animal experimentation

**DOI:** 10.3389/fnut.2025.1612746

**Published:** 2025-07-16

**Authors:** Zhujin Song, Yuhua Wu, Liping Luo, Qingqing Hu, Saiwei Wu, Miaolian Wu, Guoqing Zhang

**Affiliations:** ^1^Department of Pharmacy, The Fourth Affiliated Hospital of School of Medicine, and International School of Medicine, International Institutes of Medicine, Zhejiang University, Yiwu, China; ^2^College of Basic Medical Science, Zhejiang Chinese Medical University, Hangzhou, China; ^3^Department of Clinical Nutrition, West China Hospital, Sichuan University, Chengdu, China

**Keywords:** quercetin, depression, bibliometrics, network pharmacology, animal experimentation

## Abstract

**Objectives:**

Accumulating clinical evidence demonstrates the therapeutic potential of Traditional Chinese Medicine (TCM) in mitigating depressive disorders. This research focuses on quercetin, a principal bioactive constituent shared among five classical TCM antidepressant formulations, to systematically decode its multi-target mechanisms via an integrative framework combining neuroinflammatory modulation and synaptic plasticity regulation.

**Methods:**

A tripartite experimental design was implemented. Firstly, bibliometric analysis systematically screened antidepressant TCM prescriptions and their bioactive components. Secondly, network pharmacology delineated the therapeutic mechanisms of quercetin – a key phytochemical identified through prior analysis. Finally, we established a chronic unpredictable mild stress (CUMS)-induced depression-like behavior model in mice for validation.

**Results:**

Bibliometric analysis showed that the clinical efficacy of 5 TCM antidepressant prescriptions were identified by evidence-based medicine. In these prescriptions, *Radix Bupleuri*, *Rhizoma Cyperi*, and *Radix Glycyrrhizae* were the most commonly used herbs, while Quercetin was identified as the shared bioactive nexus across these prescriptions. Network pharmacology analysis revealed that quercetin may be closely related to PI3K/AKT pathway in depression. And results of animal experimentation showed that quercetin could improve depression-like behaviors and restore neurotransmitters levels. Concurrently, quercetin may inhibit neuroinflammation and ameliorate synaptic ultrastructural by PI3K/AKT pathway.

**Conclusion:**

The present study elucidated the mechanism of quercetin, an active ingredient in TCM prescriptions, in the treatment of depression through data mining, network pharmacology prediction, and experimental validation. This integrated research method will provide a new perspective for the development of TCM.

## Introduction

Depression refers to a neuropsychiatric disorder characterized by low mood, guilt and hopelessness. Currently, depression become the fourth leading cause of disability in the world and the incidence of depression is 4% ~ 6% of the population in China (1, 2). The pathogenesis of depression remains incompletely understood and several theoretical hypotheses have been proposed, including the neurotransmitter hypothesis, the immunoinflammatory hypothesis, neurotrophic hypothesis and the hypothalamic–pituitary–adrenal (HPA) axis hyperactivity hypothesis (3–6). Although these hypotheses have been partially corroborated, the therapeutic efficacy is still unsatisfactory. The UKs National Institute for Health and Care Excellence guidelines recommend selective serotonin reuptake inhibitor (SSRI) as the first-line treatment for people with persistent subthreshold depressive symptoms or mild to moderate depression (7). Nevertheless, some side effects associated with SSRI, including ejaculation disorder, tremor, decreased appetite and blurred vision, affect the willingness of patients to treat depression (8). Therefore, it is necessary and urgent to develop effective and safe agent to improve depression.

Traditional Chinese medicine (TCM) emphasizes the ideology of the integration of body and spirit, which considers people’s spirit, emotion and body as an interconnected whole. In this research, we applied bibliometrics and data mining methods to explore effective herbs and valuable ingredients. Quercetin, a low molecular weight flavonoid that can penetrate the blood–brain barrier, has been identified as a possible active ingredient through data mining (9, 10). Mounting studies illustrated that quercetin possesses multiple pharmacological and immunomodulatory activities including anti-inflammation, anti-oxidation, anti-ischemic, and anticancer (11–14). Currently, quercetin has been shown to exert neuroprotective effects in many neurological diseases, such as epilepsy, Alzheimer’s disease, Parkinson’s disease and cerebral ischemia/reperfusion damage (15–18).

We applied a progressive analysis in this study. Bibliometrics are applied to quantitatively and qualitatively evaluate scientific studies, and further explore the meaningful research direction (19). Subsequently, network pharmacology predicted quercetin’s putative antidepressant target. We adopted a chronic unpredictable mild stress (CUMS) model, which was first reported by Katz and colleagues in the early 1980s. They found that under multiple stimulates of unpredictable micro-stressors, the rodents exhibit anhedonia symptom, such as decreased consumption of sucrose water (20). At present, hundreds of studies have applied the CUMS model, which shows that the CUMS procedure has been accepted by most researchers. In order to deeply understand the mechanism of effective ingredient of TCM in alleviating depression, we integrated these methods to conduct research.

## Materials and methods

### Data analysis based on the bibliometrics

In this study, a systematic literature search was conducted using the Web of Science Core Collection database (2012–2022) with a combined search strategy: (“Chinese medicine” OR “traditional Chinese medicine”) AND (“depression” OR “depressive” OR “antidepressant”) NOT (animal OR mice OR rat), yielding 3,889 initial records. After dual screening of titles and abstracts to exclude non-clinical studies, case reports, and literature unrelated to TCM formulas, 127 studies were retained. A subsequent full-text review further excluded randomized controlled trials that lacked detailed formula compositions or had insufficient sample sizes. This process identified 10 high-frequency TCM formulas, among which 5 formulas were validated for clinical antidepressant efficacy through large-scale RCTs. Methodological quality of the included studies was assessed using the Cochrane Risk of Bias Assessment Tool, while meta-analysis procedures adhered to PRISMA guidelines. Two researchers independently performed literature screening and data extraction, with discrepancies resolved through group discussion or third-party expert arbitration. A “herb-formula-component” network was constructed, leading to the identification of quercetin as the core active component. Visualization of high-frequency formula clusters and evidence hierarchy analysis were performed using VOSviewer software (version 1.6.15) to ensure credibility and reproducibility of the findings.

### Screening for potential targets of depression

We chose “depression” “depressive disorders” and “antidepressant” as the retrieval terms to collect the potential targets from five databases, including OMIM database^1^, GeneCards^2^, DisGeNET^3^, Therapeutic Target Database^4^, and DrugBank^5^.

### Predicted targets of quercetin

SwissTarget Prediction database^6^ (version 2021.04), SEA^7^, STITCH^8^ (version 5.0), and PharmMapper database^9^ (version 3.0) were used to predict the targets of quercetin with the following parameters: SwissTarget Prediction confidence ≥0.7, STITCH interaction score ≥0.4, PharmMapper *Z*-score ≥2.0, SEA *E*-value <1e-5. The UniProt database (release 2022_03) was applied to standardize these targets.

### PPI network construction and hub targets analysis

The shared targets of quercetin and depression were used to construct a protein–protein interaction (PPI) network in String Database and *Homo sapiens* was selected as the target organism. PPI network can reflect the relationship between proteins. Then, we utilized Cytoscape plugin CytoHubba to perform topological analysis on the four dimensions of MNC, MCC, Degree and Betweenness centrality. Through intersecting the top 15 targets in the four dimensions, the common targets were defined as the hub targets to further study.

### Enrichment analysis and functional prediction

Gene ontology (GO) annotation and Kyoto Encyclopedia of Genes and Genomes (KEGG) pathway enrichment analysis were performed to predict the potential functions and pathways using the Comparative Toxicogenomics Database (CTD^10^), which is a robust, publicly available database integrated with functional and pathway data (21). In order to explore the functional interactions of biological process, the Cytoscape plug-in ClueGO and Cluepedia was applied.

### Animal and treatment

Male C57BL/6 mice (3-month-old, body weight: 22-25 g) were from the Experimental Animal Central of Zhejiang Chinese Medical University (GLE: cma 001756). The mice were maintained in the 12 h light/dark cycle cage, with 50–60% relative humidity and 22 ± 2° C temperature. Except during the period of food and water deprivation required by the CUMS protocol or before the sucrose preference test (SPT) assessment, mice can be assured that they have a standard diet and water at all the time. All experiment procedures were approved by the ethics committee of Zhejiang Chinese Medicine University (Hangzhou, China) (IACUC-20210719-05).

Forty mice were habituated for 7 days and then divided into five groups by random number table method, including control group, control + quercetin group, CUMS group, CUMS + quercetin (30 mg/kg/d) group, and CUMS + Fluoxetine (10 mg/kg/d) group. The dosage of quercetin was referenced from the previous research and it is safe to human (22–24). The mice in the control and control + quercetin groups were housed with 4 mice per cage, while the mice in the other three groups were housed individually. The administration was done at 9:30 a.m.

### CUMS-induced depression model

In the CUMS procedure, three stimulation methods were randomly selected each day from a predefined list of eight stressors. The randomization process was performed using a computer-generated sequence (MATLAB R2021a) to ensure non-consecutive repetition (no stimulus or stimulus combination was repeated on consecutive days) and balanced frequency (Each stressor was applied approximately equally throughout the 28-day protocol).

The eight stimulation methods included: (1) food deprivation (24 h), (2) water deprivation (24 h), (3) restraint in a 50-ml tube (2 h), (4) tail clipping (10 min), (5) exposure to a wet cage (24 h), (6) swimming in cold water (4°C for 5 min), (7) foreign odor exposure (2 h), and (8) cage shaking (10 min).

### General status and behavioral tests

We adopted the body weight to measure general status of mice and the data was recorded once a week. Anhedonia is the core symptom of depression, and SPT can be used as an indicator of anhedonia. Decreased survival desire is the most prominent feature of depression, and prolonged immobility time in tail suspension test (TST) and forced swimming test (FST) is the indicator of decreased survival desire. Therefore, we used SPT, TST and FST as behavioral indicators to observe the depressive state of mice.

During the adaptation, mice were trained to drink sugar water (1% sucrose = 1 g sucrose + 100 mL water). During the CUMS, SPT was performed after water deprivation and fasting every week, and consumptions of sucrose and water were recorded. SPT (%) = sucrose consumption/(sucrose consumption + water consumption) (25).

Behaviors in the TST and FST were recorded by Supermaze software (Shanghai Xinyuan Information Technology, China). In the TST, mice moved freely for 360 s. Immobility time during the last 240 s was quantified (26). The mice could rest for 24 h, and then mice swam for 360 s in the FST, the water temperature was maintained at 22 ± 2°C. Immobility time during the last 240 s was quantified (26).

### Western blotting and ELISA

The hippocampus was disintegrated in protein lysates solution (RIPA: PMSF = 100:1) and centrifuged at 12,000 rpm for 30 min under 4°C. Then, collecting the supernatant and testing the concentration of each protein sample by BCA assay kit. Total proteins samples were separated by sodium dodecyl sulfate polyacrylamide gel electrophoresis (12% gels) and transferred to PVDF membranes. After blocking with 5% fat-free milk for 2 h at room temperature, the membranes were incubated overnight at 4°C with rabbit antibodies against: PI3K (1:1000; CST), p-PI3K (1:1000; Abcam), AKT (1:1000; CST), p-AKT (1:1000; CST), GFAP (1:1000; Proteintech), Iba1 (1:1000; CST), TNF (1:1000; Proteintech), IL-1β (1:1000; CST), PSD95 (1:1000; CST), and Arc (1:1000; Synaptic Systems). Next, we washed with PBST (3 × 10 min) and the membranes were incubated with the horseradish peroxidase (HRP)-conjugated secondary antibodies at room temperature for 2 h. To maximize antibody binding efficiency, each membrane was strategically cut into strips before blocking/incubation. The antigen–antibody complex was scanned by the Chemistar™ High-sig ECL Western Blotting Substrate (Tanon, China). And all strips were incubated with identical antibody batches, and the exposure time for the same protein was maintained consistently. Band intensities were densitometrically quantified using ImageJ software (Version 1.8.0, NIH, United States).

Blood was collected immediately after the mice were sacrificed. After 20 min at room temperature and then centrifuged for 20 min (3,000 rpm), the supernatant was obtained. According to the instructions of the ELISA kits, the levels of 5-HT, norepinephrine, TNF-*α* and IL-1β in serum and hippocampus were measured.

### Immunofluorescence and golgi staining

The brains of the mice were removed and fixed in 4% paraformaldehyde for 24 h, then the brains were transferred to 30% sucrose solution at 4°C for 24 h. Brain slices were set as 10 μm and blocked in 0.1 M PBS containing 10% goat serum and 0.4% Triton X-100 for 1 h, then incubated overnight at 4°C in the corresponding primary antibodies: GFAP (1:100; Proteintech) and Iba1 (1:100; CST) were incubated with Brain slices overnight at 4°C. After washing with PBS (3 × 15 min), the slices were incubated with FITC-labeled anti-rabbit IgG and TRITC-labeled anti-mouse IgG for 2 h. Staining DAPI (4′,6-diamidino-2-phenylindole) was conducted in the final 10 min. Fluorescent images were obtained using LSM710 Confocal Laser Scanning Microscope (Zeiss, Germany).

After brains were fixed, the hippocampal regions were cut into 2–3 mm tissue blocks, and the tissue blocks were washed 3 times using saline. After washing, dye solution (Service Bio, G1069) was added and left for 14 days in a dark room. The staining solution was changed every 72 h. After 14 days, tissue blocks were removed from the staining solution and placed in 15% sucrose for 24 h (4°C, dark room). After 24 h, the tissue blocks were removed and placed in 30% sucrose for 24–36 h (4°C, dark room). After dehydration ended, the tissue pieces were washed and then soaked by adding concentrated ammonia water. The tissue blocks were soaked using acidic fixative for 45 min. Tissue blocks were rehydrated using 30% sucrose water (4°C, dark room, 48–72 h). Frozen sections were completed and then were scanned using a digital section scanner (D HISTECH, Pannoramic 250).

### Statistical analysis

Data were represented as the means ± SEM and analyzed using GraphPad Prism 8.0 (GraphPad Inc., San Diego, CA, United States). Behavioral tests and neurotransmitter levels were compared by one-way ANOVA followed by Tukey’s *post hoc* test. Protein expression data were analyzed with Kruskal–Wallis test followed by Dunn’s test with Bonferroni correction. A value of *p* < 0.05 (adjusted for multiple comparisons where applicable) was considered significant.

## Results

### Distribution of TCM prescriptions

**Figure 1:**
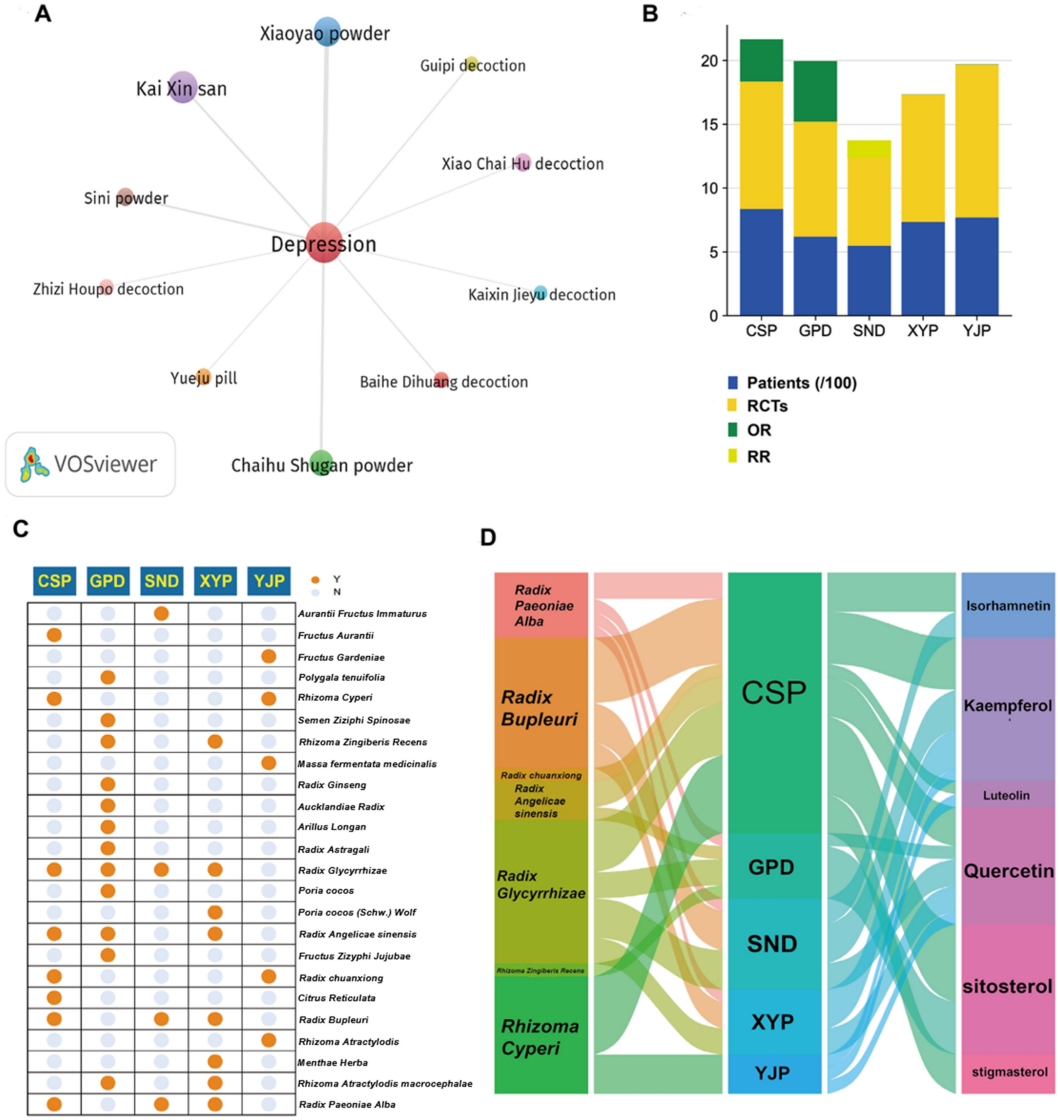
Bibliometric profiling of clinically effective TCM prescriptions for depression treatment. **(A)** VOSviewer co-occurrence analysis identifies 10 high-frequency TCM prescriptions for depression treatment. **(B)** Evidence-based medicine validation confirms clinical efficacy of five TCM prescriptions (CSS, GPD, SNS, XYS, YJP). **(C)** Twenty-four constituent herbs within the five validated prescriptions are cataloged. **(D)** Seven core herbs and six bioactive compounds demonstrate cross-prescription prevalence with quercetin as the central shared component.

A total of 3,889 studies were published in journals. Screened by title and abstract, there were 127 studies referring to TCM prescriptions in the treatment of depression. Among them, 10 TCM prescriptions, including Baihe Dihuang decoction (BDD), Chaihu Shugan San (CSS), Guipi decoction (GPD), Kai Xin San (KXS), Sini San (SNS), Xiaoyao San (XYS), Zhizi Houpo decoction (ZZHPD), Kaixin Jieyu decoction (KXJYD) and Yueju pill (YJP), were identified as high-frequency prescriptions and research hotspots by VOSviewer software (Figure 1A). Through searching these TCM prescriptions, we found that the efficacy of CSP, GPD, SND, XYP, YJP has been confirmed by a large number of clinical randomized double-blind controlled trials (RCTs). Figure 1B shows the results of evidence-based medicine (EBM) studies for the five TCM prescriptions, which are considered to represent the highest level of evidence (27). Among these prescriptions, Radix Bupleuri, Rhizoma Cyperi, Radix Glycyrrhizae, Radix Paeoniae Alba, Radix Angelicae sinensis, Radix chuanxiong, and Rhizoma Zingiberis Recens were the most commonly used herbs, while quercetin, kaempferol, luteolin, stigmasterol, isorhamnetin, and sitosterol were the most usual ingredients (Figures 1C,D). Since the effect of quercetin on neurological diseases has been partially confirmed, we believe that quercetin may have important potential and research value in the treatment of depression.

### Identification the hub targets of quercetin against depression

**Figure 2:**
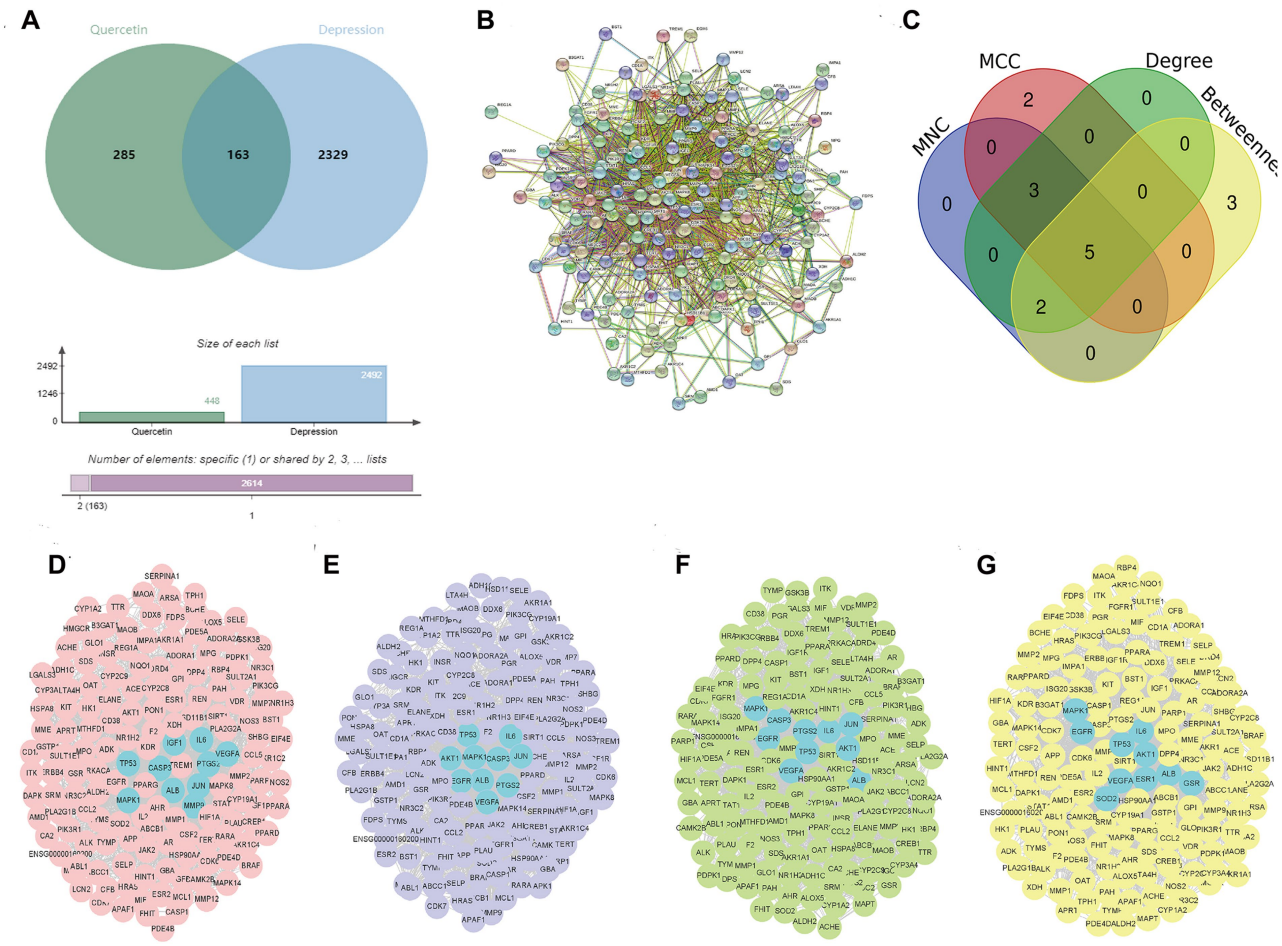
Identification of Hub targets for quercetin in depression. **(A)** Venn diagram of 163 shared targets between depression (2,492 targets) and quercetin (448 targets). **(B)** PPI network of shared targets (162 nodes, 1741 edges; STRING database). **(C)** MNC algorithm top targets. **(D)** MCC algorithm top targets. **(E)** Degree centrality top targets. **(F)** Betweenness centrality top targets. **(G)** Ten intersected hub targets (VEGFA, ALB, IL6, MAPK1, TP53, JUN, CASP3, PTGS2, AKT1, EGFR).

We obtained 2,492 targets related to depression and 448 predicted targets of quercetin through databases. The Venn diagram identified 163 shared targets between quercetin and depression (Figure 2A) and a PPI network with 162 nodes and 1741 edges were constructed by String database (Figure 2B). According to the results of MNC, MCC, Degree and Betweenness, there were 10 intersected targets, including VEGFA, ALB, IL6, MAPK1, TP53, JUN, CASP3, PTGS2, AKT1, and EGFR (Figures 2C–G), were identified as the hub targets.

### GO and KEGG enrichment analysis

**Figure 3:**
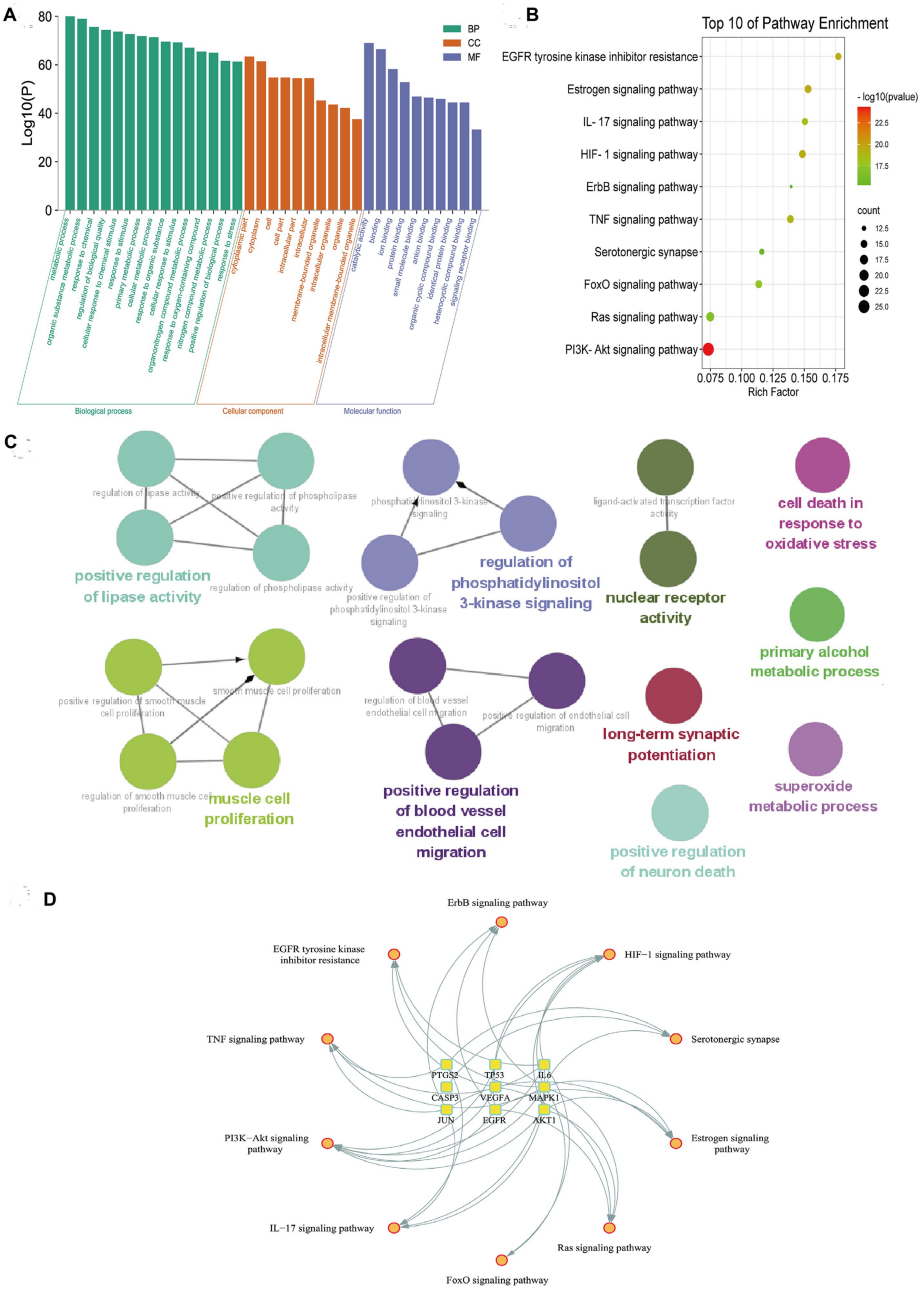
Functional enrichment analysis of quercetin’s shared targets in depression. **(A)** Gene Ontology enrichment shows dominant biological processes, cellular components, and molecular functions. **(B)** KEGG pathway analysis ranks PI3K/AKT signaling as the most significant pathway. **(C)** ClueGO network visualization associates targets with neuroinflammation and synaptic regulation terms. **(D)** Hub target-pathway mapping demonstrates convergence of six targets on PI3K/AKT signaling.

GO enrichment showed that metabolic process, response to stress, and biological regulation were the main part in biological processes (BP). As for BP, intracellular space is obvious. The results of molecular functions (MF) emphasized protein binding and signaling receptor binding (Figure 3A). The most valuable pathways obtained by KEGG analysis were PI3K/AKT signaling pathway, TNF signaling pathway, FoxO signaling pathway, and Ras signaling pathway (Figure 3B). And the results of ClueGO analysis showed that the main enriched terms were regulation of phosphatidylinositol 3-kinase signaling, long-term synaptic potentiation, positive regulation of neuron death, and superoxide metabolic process (Figure 3C). Among them, the PI3K/Akt pathway is the most significant, which is consistent with the analysis results of KEGG. Meanwhile, the “pathways-targets” network showed that the PI3K/AKT signaling pathway enriched the most hub targets, including AKT1, EGFR, IL6, MAPK1, TP53, and VEGFA (Figure 3D).

### Quercetin alleviates depression-like behaviors and regulates the level of 5-HT and NE in CUMS mice

**Figure 4:**
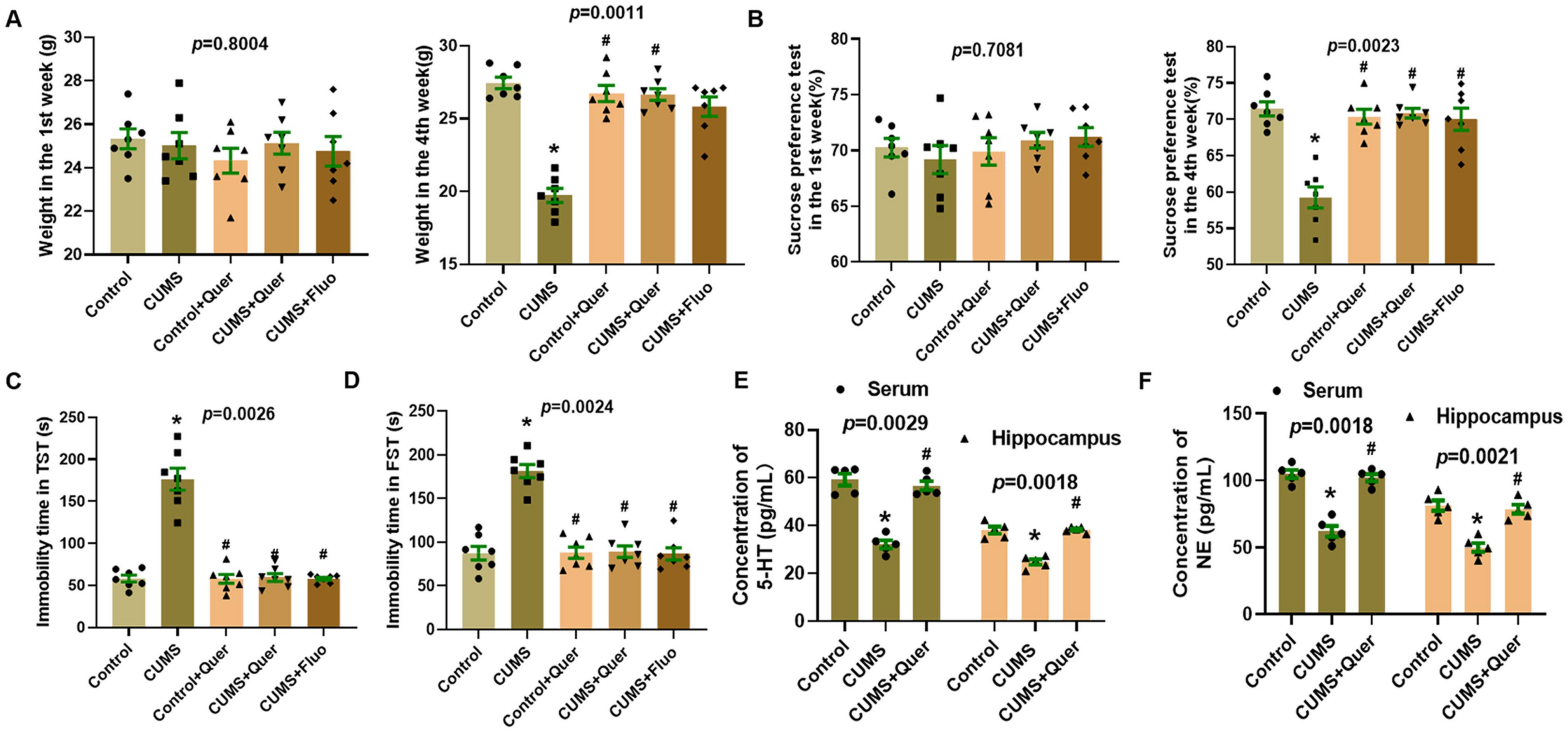
Quercetin alleviated depression-like behaviors and the neurotransmitters levels in CUMS mice. **(A)** Body weights in the 1st week and 4th week (*n* = 7). **(B)** Sucrose solution intake in the 1st week and 4th week (*n* = 7). **(C)** Immobility time in tail suspension test (*n* = 7). **(D)** Immobility time in forced swim test (*n* = 7). **(E)** The levels of 5-HT in serum/hippocampus were determined by ELISA (*n* = 5). **(F)** The levels of NE in serum/hippocampus were determined by ELISA (*n* = 5). ^*^p < 0.05, vs. Control group. ^#^*p* < 0.05, vs. CUMS group.

Following 4 weeks of CUMS procedure, the body weights of the mice in CUMS group were significantly lower than those in control group. And administration with quercetin markedly reversed this phenomenon (Figure 4A; Supplementary Table S1; Supplementary Figure S1). As for the behavioral tests, the results of SPT showed that CUMS procedure significantly reduced the sucrose intake of mice in CUMS group compared with in control group (Figure 4B). The immobility time in TST and FST of mice in CUMS group had significantly increased compared to the mice in control group. The results of behavioral tests indicated the successful establishment of the depression-like behaviors model. Based on this model, we further evaluated the effect of quercetin in ameliorating CUMS-induced depression-like behaviors in mice. The results showed that compared with CUMS group, the SPT index was increased after quercetin administration, and the immobility times in TST and FST were reduced (Figures 4C,D).

We also evaluated the level of 5-HT and NE in serum and hippocampus. The results showed that compared with control group, the levels of 5-HT and NE in CUMS group were significantly decreased, whereas treatment with quercetin make it return to the normal level (Figures 4E,F). These results implied that quercetin can alleviate depression-like behaviors and regulate the level of 5-HT and NE in CUMS mice.

### Quercetin inhibits the activation of glial cells and neuroinflammation in CUMS mice

**Figure 5:**
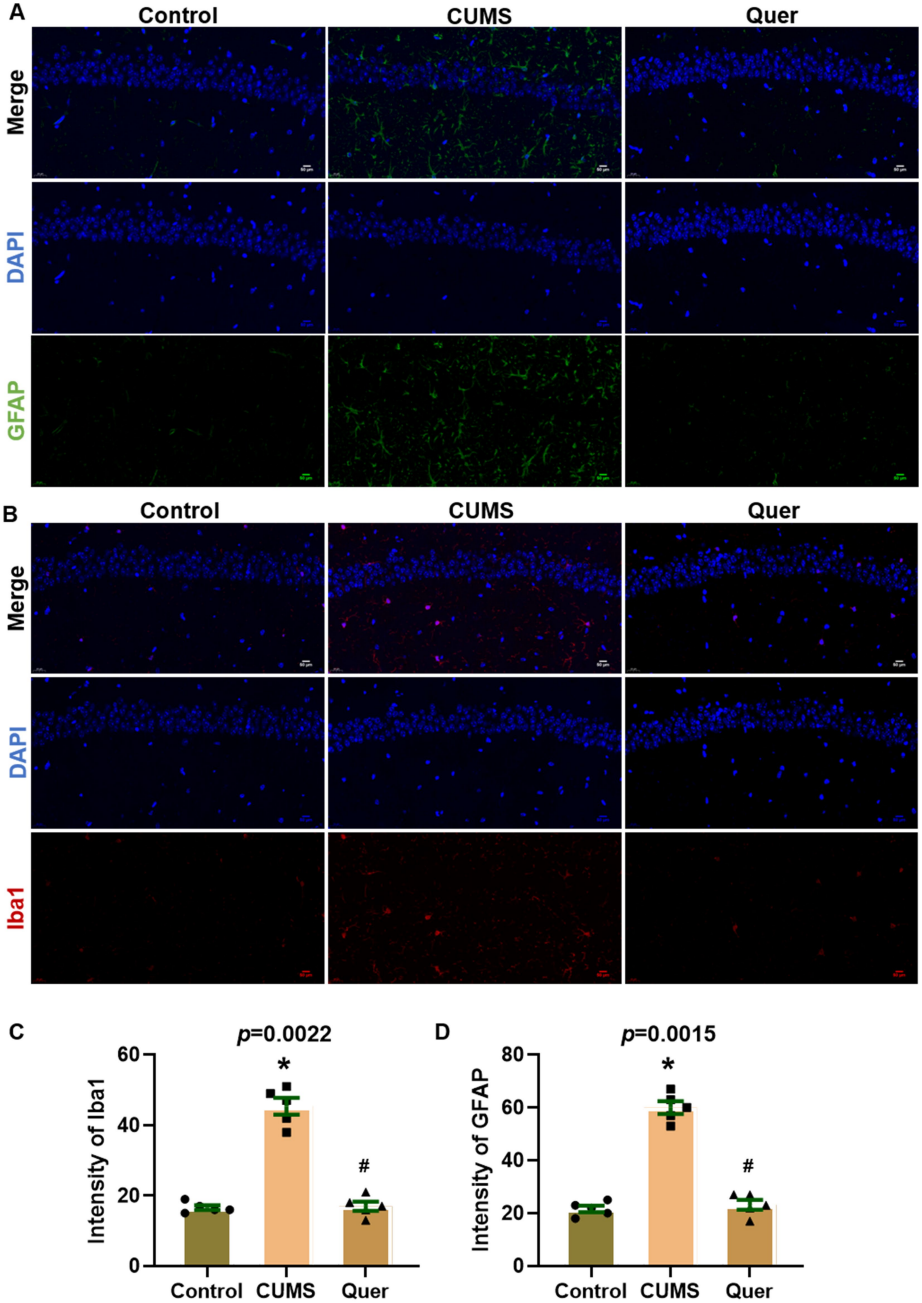
Quercetin suppressed glial activation in hippocampal CA1 of CUMS mice. **(A)** Representative immunofluorescence images for GFAP (green) in hippocampal CA1 region, showing increased intensity in CUMS mice compared to Controls, which was reduced by quercetin treatment. **(B)** Representative immunofluorescence images for Iba1 (red) in CA1 region, revealing elevated signal in CUMS mice that was attenuated by quercetin. **(C,D)** Quantitative analysis of GFAP and Iba1. Scale bar: 50 μm; DAPI (blue). ^*^p < 0.05, vs. Control group. ^#^*p* < 0.05, vs. CUMS group.

**Figure 6:**
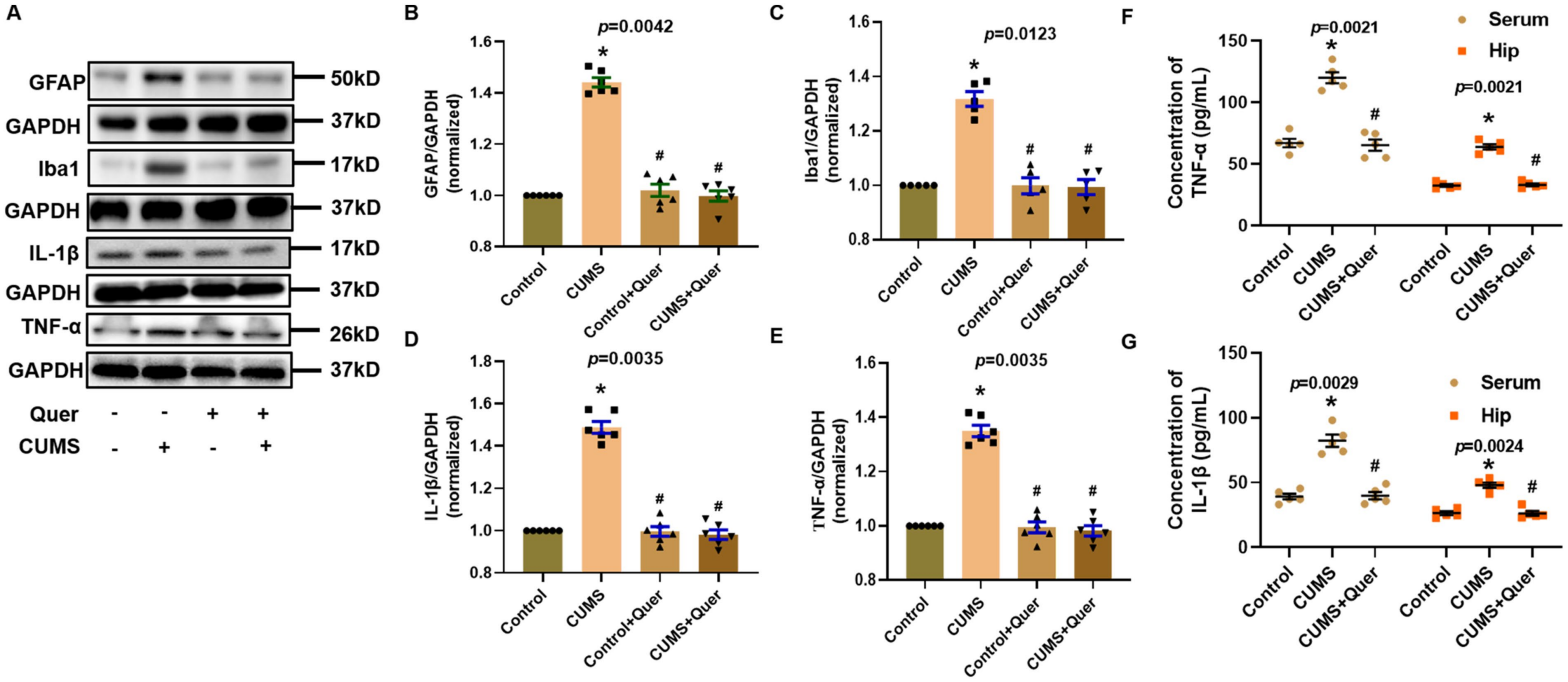
Quercetin alleviated neuroinflammation induced by CUMS in hippocampus. **(A)** Representative blots of GFAP, Iba1, IL-1β and TNF-*α*. **(B,C)** The levels of GFAP (*n* = 6) and Iba1 (*n* = 6) were analyzed, and the protein quantification confirmed quercetin’s inhibitory effect on glial activation. **(D,E)** The levels of IL-1β (*n* = 6) and TNF-α (*n* = 6) were analyzed, and shows suppression of inflammatory cytokines by quercetin. **(F,G)** ELISA quantification of TNF-α (*n* = 5) and IL-1β (*n* = 5) levels in hippocampal tissue and serum. ^*^p < 0.05, vs. Control group. ^#^*p* < 0.05, vs. CUMS group.

Network pharmacology predicted that quercetin against depression was associated with the inflammatory response. Astroglial and microglial activation constitutes a fundamental pathomechanism underlying neuroinflammatory cascades. Immunofluorescence quantification revealed elevated hippocampal GFAP (astrocyte marker) (Figures 5A,D) and Iba1 (microglial marker) (Figures 5B,C) expression in CUMS-exposed mice relative to controls. The result of WB was also consistent with IF staining (Figures 6A–C). We also detected the cytokine levels, including TNF-α and IL-1β, in the hippocampus by WB and ELISA. Complementary assessments of neuroinflammatory mediators demonstrated dose-dependent suppression of TNF-α and IL-1β levels via both western blotting (Figures 6D,E) and ELISA quantification (Figures 6F,G). Collectively, these multimodal data substantiate quercetin’s capacity to mitigate CUMS-induced neuroinflammation through dual regulation of glial reactivity and cytokine production.

### Quercetin improves synaptic damage in the hippocampus of CUMS mice

**Figure 7:**
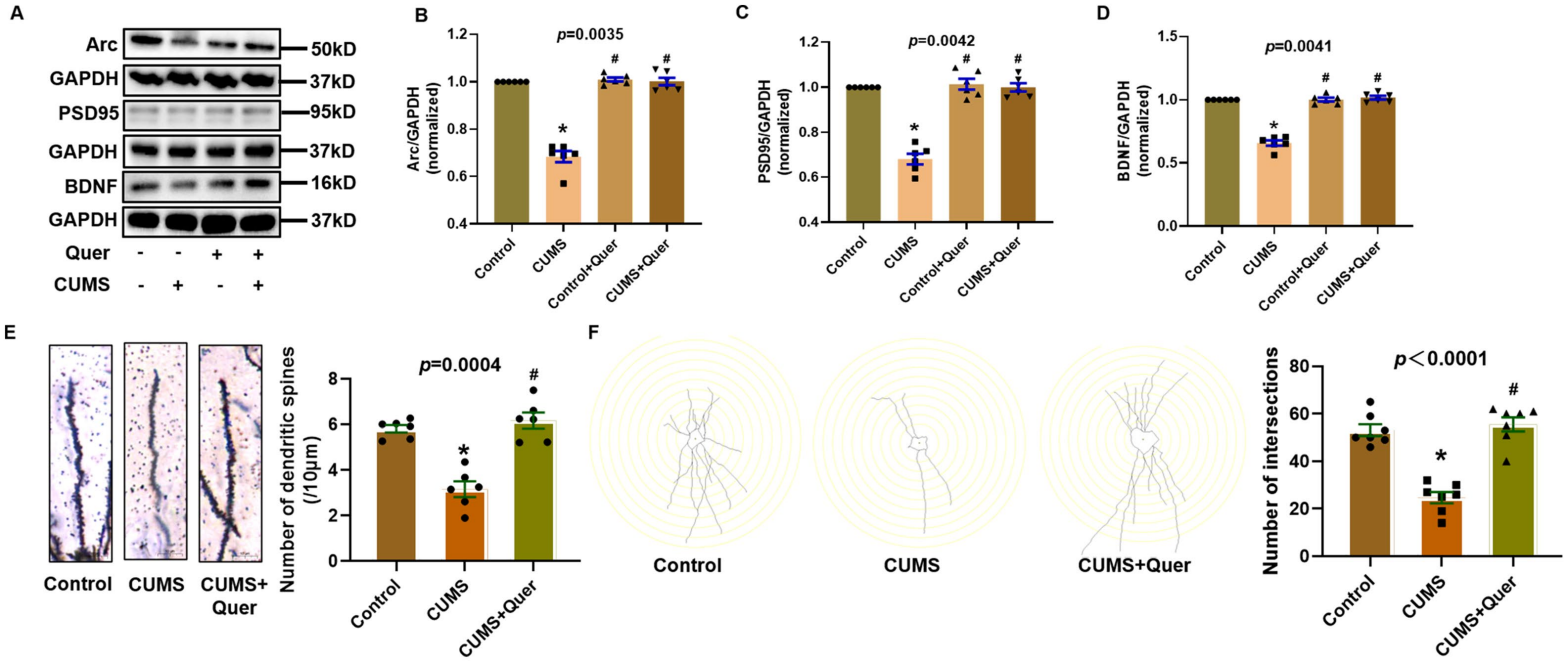
Quercetin improves synaptic damage in the hippocampus of CUMS mice. **(A)** Representative blots of Arc, PSD95 and BDNF. **(B–D)** Quantification of Arc (*n* = 6), PSD95 (*n* = 6) and BDNF (*n* = 6) were analyzed, and showing significant increases following quercetin treatment. **(E)** Dendritic spine density quantification in CA1 (*n* = 6), demonstrating quercetin-mediated restoration of spine density. **(F)** Synaptic structural analysis in CA1 (*n* = 7), confirming quercetin-induced repair of synaptic ultrastructure. ^*^p < 0.05, vs. Control group. ^#^*p* < 0.05, vs. CUMS group.

PSD95 and Arc are the important marker proteins of synaptic plasticity, which has been demonstrated to be closely involved in depression. In this study, PSD95, Arc and BDNF were shown to have lower expression in CUMS group than that in control group, which indicated that CUMS procedure affected the synaptic plasticity of mice hippocampal neurons. Pharmacological intervention with quercetin (30 mg/kg/d) effectively restored these synaptic biomarkers to near-physiological levels (Figures 7A–D), demonstrating therapeutic potential against CUMS-mediated synaptic dysfunction. Complementary morphological analyses through golgi staining quantitatively validated these findings, with quercetin-treated cohorts exhibiting increased dendritic spine density and enhanced synaptic length in CA1 region (Figures 7E,F), thereby providing ultrastructural evidence for synaptic integrity preservation.

### Quercetin stimulates the PI3K/AKT pathway in CUMS mice

**Figure 8:**
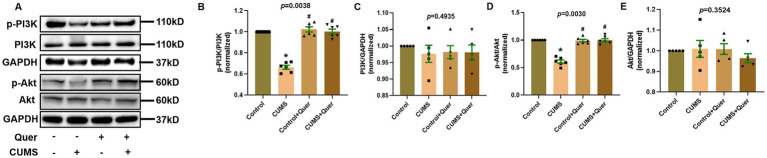
Quercetin stimulates the PI3K/AKT pathway in the hippocampus of CUMS mice. **(A)** Representative blots of p-PI3K, p-Akt, PI3K and Akt. **(B,C)** The levels of p-PI3K (*n* = 6) and PI3K (*n* = 6) were analyzed, and quercetin administration enhanced PI3K phosphorylation. **(D,E)** The levels of p-Akt (*n* = 6) and Akt (*n* = 6) were analyzed, and quercetin administration enhanced Akt phosphorylation. ^*^p < 0.05, vs. Control group. ^#^*p* < 0.05, vs. CUMS group.

According to the pathway enrichment analysis, the PI3K/AKT signaling pathway was predicted to be one of the most significant pathways, which involved in six hub targets (AKT1, EGFR, IL6, MAPK1, TP53, and VEGFA). However, the results of network pharmacology can only give hints, which still need to be verified by actual experiments. In animal experiment, the expression of p-PI3K and p-AKT were significantly decreased in CUMS group compared to control group. After quercetin treatment, the expression of p-PI3K and p-AKT were significantly increased (Figures 8A,B,D). Furthermore, there was no significant difference in total-PI3K and total-AKT among these groups (Figures 8A,C,E). The above presented results suggested that quercetin improved CUMS induced depression-like behaviors via the PI3K/AKT pathway.

## Discussion

Thousands of years of clinical practice have proved that Chinese herbs have better clinical efficacy and fewer side effects in protecting people from depression. According to bibliometric analysis, quercetin was identified as the potential ingredient in alleviating depression. Network pharmacology and bioinformatics results predicted that quercetin amelioration of depression may be related to neuroinflammation. The signaling pathways mainly included PI3K/AKT pathway and TNF pathway. It is well known that the central nervous system (CNS) is a complex regulatory system and has complex neuronal network. Bioinformatics and network pharmacology can only provide us with a prediction and hypothesis; the animal experimental verification is important and essential. Indeed, this prediction was strongly supported by subsequent animal experiments.

Monoamine neurotransmitters, such as 5-HT and NE, are the chemical messengers to transmit neurological signal. A considerable amount of research has demonstrated that the decreased 5-HT content in the brain are closely related to depression (28). Most researchers support that the deficiency of neurotransmitters is the major pathogenesis of depression (29). Currently, SSRI fluoxetine hydrochloride has been widely used as the first-line regimen for depression (30). However, 30–40% of patients with depression are unresponsive to fluoxetine treatment. In order to clarify the effects of quercetin on neurotransmitters, we measured the levels of 5-HT in the hippocampus and serum. The results indicated that both in the hippocampus and serum, the CUMS procedure significantly decreased the levels of 5-HT, and quercetin reversed this phenomenon. Previous studies confirmed that the regulation of 5-HT levels by quercetin is partly due to inhibit the monoamine oxidase-A (MAO-A), which affects the metabolism of 5-HT in the brain (31, 32). In addition to 5-HT, NE levels in the hippocampus and serum also play a significant role in depression. According to literatures, decreased of NE level of hippocampus produced depression-like behaviors whereas increased NE level alleviated these behaviors (33–35). A systematic review revealed that the addition of serotonin and norepinephrine reuptake inhibitors (SNRI), such as venlafaxine and duloxetine, to patients with major depression who have failed to reach remission with a SSRI can achieve good clinical effects (36). Therefore, quercetin, both increasing 5-HT and NE levels in the hippocampus and serum, holds great potential for the development of antidepressant.

Excessive neuroinflammation represents a core pathological feature of depression, establishing a bidirectional vicious cycle by disrupting neurotransmitter homeostasis, impairing synaptic plasticity, and compromising neural circuit functionality (37–40). This study demonstrates that quercetin counteracts this process through multi-tiered interventions. Regarding neuroinflammation, quercetin not only normalizes elevated levels of hippocampal and serum pro-inflammatory cytokines (TNF-*α*, IL-1β) in the CUMS model but also significantly suppresses activation of astrocytes (GFAP) and microglia (Iba1). In CNS, activated astrocytes and microglia are the main sources of inflammatory cytokines (41, 42). These effects converge with previously established inhibition of NF-κB/AP-1 signaling pathways (43) and blood–brain barrier preservation—specifically through maintaining connexin expression to block peripheral inflammatory cell infiltration (44)—collectively forming a concerted anti-inflammatory network. At the synaptic plasticity level, quercetin effectively reverses CUMS-induced downregulation of PSD95 and Arc expression. This restoration arises both from inhibiting microglia-mediated synaptic elimination (45, 46) and through direct repair of synaptic ultrastructure, thereby disrupting the “neuroinflammation-synaptic impairment” cycle.

According to the prediction results of network pharmacology, PI3K/AKT pathway was considered as the most significantly enriched pathway. In parallel, the ClueGO results also pointed to the regulation of PI3K signaling. PI3K/AKT pathway is a widespread cellular transduction pathway that plays a role in regulating cell metabolism and immune system function (47). PI3K is an intracellular phosphatidyl-inositol kinase that can specifically catalyze PIP2 to PIP3. PIP3 facilitates the activation of AKT by binding to the PH domain of AKT and this may cause neuroinflammation (48). And we found that quercetin could improve neuroinflammation and synaptic plasticity. On the inflammatory front, by reducing TNF-α/IL-1β release and glial cell activation—potentially via PI3K/AKT-mediated suppression of NF-κB nuclear translocation—it terminates inflammatory damage to synapses. Conversely, on the synaptic front, activation of the PI3K/AKT–mTOR axis promotes synaptic protein (PSD95/Arc) translation and dendritic spine remodeling, with our observed restoration of synaptic biomarkers supporting this mechanism. Such bidirectional regulation enables quercetin to cooperatively interrupt the neuroinflammation-synaptic dysfunction vicious cycle. The potential role of downstream mediator GSK-3β—implicated in tau phosphorylation and apoptosis—requires further validation. Moreover, researchers have certified that AKT activity in the brain of depressed suicide victims was attenuated (49, 50). As analytically predicted above, quercetin administration significantly increased the levels of p-PI3K and p-AKT in the hippocampus. However, the levels of t-PI3K and t-AKT were not changed, indicating that only the phosphorylation levels of these proteins were affected.

The results of this study suggested the antidepressant effects of quercetin were mainly due to increasing the levels of 5-HT and NE in hippocampus and serum, inhibiting the activation of glial cells and neuroinflammation, and improving the synaptic plasticity of hippocampal neurons, which might be associated with PI3K/AKT signaling pathway. Our study indicated that quercetin could be a potentially promising therapeutic option or an adjuvant drug for depression. Moreover, quercetin, as a common dietary supplement, has good safety (51). Although limited oral bioavailability presents challenges, nanocarrier-based delivery approaches offer a viable pathway for clinical translation of this multi-target natural compound (52).

## Conclusion

In summary, we screened an active ingredient quercetin from clinically effective TCM prescriptions and primarily determined its pharmacological mechanisms. This study suggested that quercetin may be a promising adjuvant drug for the treatment of depression. It is worth mentioning that the integrated method in this study might provide a meaningful idea for the research and development of TCM.

## Data Availability

The original contributions presented in the study are included in the article/Supplementary material, further inquiries can be directed to the corresponding authors.

## Footnotes

^1^ http://www.omim.org

^2^ https://www.genecards.org/

^3^ https://www.disgenet.org/

^4^ http://bidd.nus.edu.sg/group/cjttd/

^5^ https://www.drugbank.ca/

^6^ http://www.swisstargetprediction.ch/

^7^ https://sea.bkslab.org/

^8^ http://stitch.embl.de/

^9^ https://www.lilab-ecust.cn/pharmmapper/

^10^ http://ctdbase.org/
