# Supplementary material for: Mechanistic insights into the anti-depressant effect of quercetin: an integrated bibliometrics, bioinformatics, and animal experimentation
Source: Front Nutr. 2025 Jul 16;12:1612746. doi: 10.3389/fnut.2025.1612746 (PMC12307201; doi:10.3389/fnut.2025.1612746)
Supplement: Supplementary file 1 [file Table_1.DOCX]

**Tab.S1 Body Weight from Week 1 to Week 4 (Mean ± SEM)**

| **Groups** | **Baseline Body Weight (g)** | | **Body Weight at 2nd Week (g)** | **Body Weight at 3rd Week (g)** | **Body Weight at 4th Week (g)** | **Body Weight Change Rate (Week 1 to 4, %)** |
| --- | --- | --- | --- | --- | --- | --- |
| **Control** | | **25.33±0.4597** | **25.30±0.4186** | **26.61±0.4014** | **27.44±0.3866** | **8.3%** |
| **CUMS** | | **25.01±0.6049** | **24.44±0.5380** | **22.73±0.6413** | **19.73±0.4799** | **-21.1%** |
| **Control+Quer** | | **24.33±0.5710** | **24.20±0.5052** | **25.40±0.4220** | **26.73±0.5562** | **9.9%** |
| **CUMS+Quer** | | **25.13±0.4994** | **24.31±0.6053** | **25.00±0.5619** | **26.64±0.4005** | **6%** |
| **CUMS+Fluo** | | **24.76±0.6778** | **25.03±0.5236** | **25.60±0.5246** | **25.81±0.6699** | **4.2%** |

**Fig.S1 Line Graph of Body Weight from Week 1 to Week 4 (Mean ± SEM)**
